# Correction: Furlan, G. and Galupa R. Mechanisms of Choice in X-Chromosome Inactivation. *Cells* 2022, *11*, 535

**DOI:** 10.3390/cells12060950

**Published:** 2023-03-21

**Authors:** Giulia Furlan, Rafael Galupa

**Affiliations:** 1 Wellcome Trust/Cancer Research UK Gurdon Institute, University of Cambridge, Tennis Court Road, Cambridge CB2 1QN, UK; 2 Department of Genetics, University of Cambridge, Downing Street, Cambridge CB2 3EH, UK; 3 Developmental Biology Unit, European Molecular Biology Laboratory, 69117 Heidelberg, Germany

The authors wish to make the following changes to their paper [1]. In the original publication, there was a mistake uploading the figures, and Figure 1, Figure 2 and Figure 3 as published do not correspond to the updated versions after peer review. In particular, Figure 1 needed to be corrected regarding the transcriptional status of the X chromosomes during the preimplantation stages. Figure 1, Figure 2 and Figure 3 should be changed to:

The authors apologize for any inconvenience caused and state that the scientific conclusions are unaffected. This correction was approved by the academic editor. The original publication has also been updated.

## Figures and Tables

**Figure 1:**
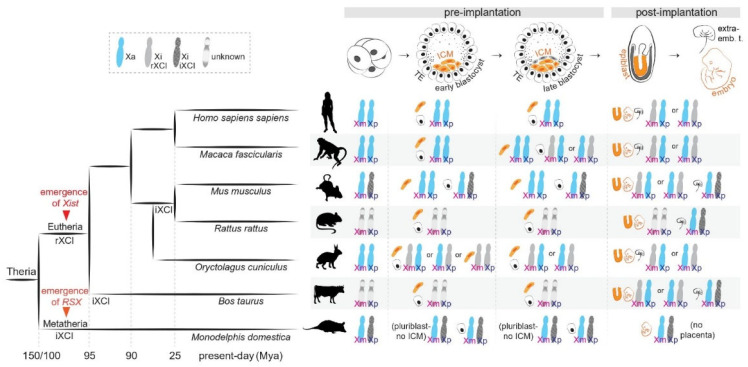
X-chromosome inactivation across species. (**Left**): Phylogenetic tree indicating the evolution of random and imprinted XCI and the emergence of long non-coding RNAs *Xist* and *RSX* in Theria. (**Right**): X-chromosome inactivation dynamics across development in representative species.

**Figure 2:**
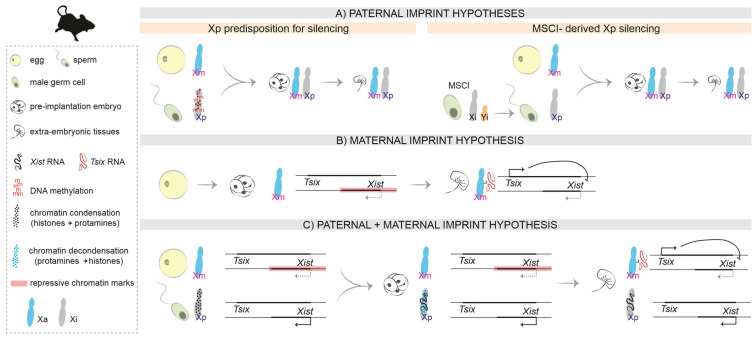
Hypotheses on the molecular nature of the imprint in mice. (**A**). Paternal imprint: The Xp inherits a predisposition for silencing from its life cycle in the male. (**B**). Maternal imprint: In the preimplantation embryo, repressive chromatin marks on the Xm (including the *Xist* promoter region) prevent *Xist* expression on the Xm. In the extra-embryonic tissues of the post-implantation embryo, *Tsix* expression prevents *Xist* upregulation in *cis*. (**C**). Paternal and maternal imprint: A combination of both hypotheses, also considering the different chromatin condensation states of the Xp in the sperm and in the paternal pronucleus after fertilization.

**Figure 3:**
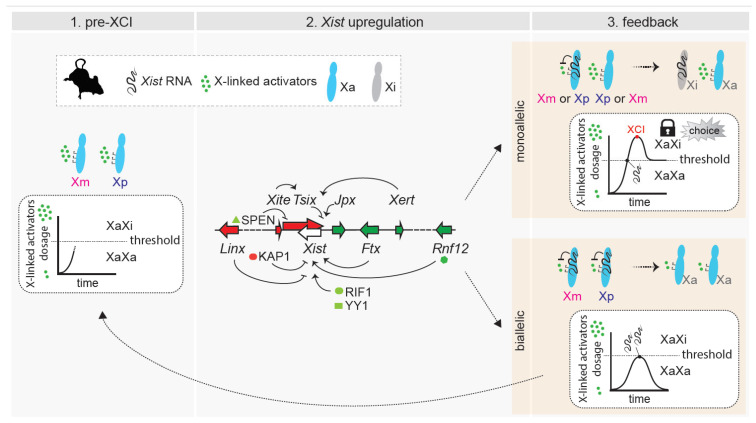
Dynamic model of choice during random XCI. (**Left**): Pre-XCI status. Both X chromosomes are active and transcribe X-linked genes. The dose of X-linked activators increases towards the threshold necessary for productive *Xist* upregulation. (**Middle**): Biallelic X-chromosome transcription allows the cell to reach the threshold for *Xist* activation. X-linked and autosomal *cis* and *trans* positive and negative regulators influence the initiation of *Xist* upregulation, which can occur on a single X chromosome or on both of them. Only factors and loci discussed in the text have been included in the figure. (**Right** (**top**)): In cells that have upregulated *Xist* monoallelically, X-wide *cis*-silencing triggered by *Xist* RNA causes a drop in the level of activators, preventing the second chromosome from upregulating *Xist*. The choice is locked in. Monoallelic *Xist* expression (and *cis*-silencing) has to be sustained through enough dosage of activators and/or feedback mechanisms. (**Right** (**bottom**)): In cells that have upregulated *Xist* biallelically, excess *Xist* expression triggers rapid downregulation of X-linked activators on both X chromosomes, and this drop in levels below the threshold causes *Xist* expression to switch off. Both X chromosomes remain active, and the process has to start again.
